# Multimorbidity in older adults: magnitude and challenges for the Brazilian health system

**DOI:** 10.1186/s12889-015-2505-8

**Published:** 2015-11-25

**Authors:** Bruno Pereira Nunes, Elaine Thumé, Luiz Augusto Facchini

**Affiliations:** Department of Nursing, Federal University of Pelotas, Rua Gomes Carneiro, 1, 96010-610 Pelotas, RS Brazil; Department of Social Medicine, Postgraduate Program of Epidemiology, Federal University of Pelotas, Pelotas, Brazil; Postgraduate Program of Nursing, Federal University of Pelotas, Pelotas, Brazil

**Keywords:** Comorbidity, Multimorbidity, Chronic diseases, Aged, Elderly, Cross-sectional studies, Brazil

## Abstract

**Background:**

Multimorbidity is a public health problem with high prevalence and important consequences. The aim of this paper was to verify the prevalence and distribution of multimorbidity in Brazilian older adults.

**Methods:**

A population-based survey was carried out in 2008 through face-to-face interviews with 1593 older adults (aged 60 or over) living in Bagé, a medium-sized city in Southern Brazil. Multimorbidity was evaluated by 17 morbidities and operationalized according to two cutoff points: 2 or more and 3 or more morbidities. Descriptive analysis examined the occurrence of multimorbidity by demographic, socioeconomic and health services variables. Observed and expected dyads and triads of diseases were calculated.

**Results:**

From total sample, 6 % did not have morbidities. Mean morbidity was 3.6. Morbidities showing higher prevalence were high blood pressure – HBP – (55.3 %) and spinal column disease (37.4 %). The percent of participants with multimorbidity was 81.3 % (95 % CI: 79.3; 83.3) for 2 or more morbidities and 64.0 % (95 % CI: 61.5; 66.4) for 3 or more morbidities. In both measures occurrence was higher among women, the more elderly, less socioeconomic status, the bedridden, those who did not have a health private plan, those who used health services and those living in Family Health Strategy catchment areas. We found 22 dyads of morbidities with prevalence 10 % or more and 35 triads with prevalence 5 % or more. The most prevalent observed pair and triplet of morbidities were HBP and spinal column disease (23.6 %) and HBP, rheumatism/arthritis/arthrosis and spinal column disease (10.6 %), respectively.

**Conclusions:**

Multimorbidity frequency was high in the sample studied, in keeping with percentage found in other countries. The social inequities identified increase the health system challenges for the management of multimorbidity, requiring a comprehensive and multidimensional care. The combinations of diseases can provide initial input to include multimorbidity in Brazilian clinical protocols.

## Background

Multimorbidity is the occurrence of multiple health problems in the same individual [1]. Its concept and operationalization have been increasingly discussed [2, 3] due to the rise – absolute and relative – in noncommunicable chronic diseases (NCD) and health expectancy of the world’s population [4, 5].

Multimorbidity is a public health problem in terms of its prevalence, severity and possibility of control [6]. The occurrence of various health problems in older adults is high (>50 %) [1] and the consequences may include increased risk of death and functional decline [7], besides having a negative impact on the quality of life and life expectancy [8].

In Brazil, despite the problem’s relevance, resulting from accelerated demographic and epidemiologic transition, studies about multimorbidity are scarce. The few studies identified addressed women aged 40 to 65 with 11 or more years of schooling [9], women ≥50 years old [10] and adults ≥18 years old as their target population [11].

Although multimorbidity is possible to control, an adequate approach to its management is a challenge for health systems and services worldwide [12]. In Brazil, the Unified Health System *(Sistema Único de Saúde – SUS)* and the Family Health Strategy (FHS) *(Estratégia Saúde da Família)* have made important progress in health service coverage and use, including the poorest and most vulnerable populations [13–15].

The FHS is the organizational axis of primary health care (PHC) in Brazil [16]. It is based on a multidisciplinary team working with a defined population and territory and has the potential to identify and monitor elderly people with NCDs and multimorbidity. However, to increases its effectiveness, FHS should improve multidimensional assessment of the elderly, in order to establish complex care plans, including prevention and health promotion, thus guiding the organization and the provision of health services [17–19].

Knowing the magnitude of multimorbidity can contribute to the organization of services, health worker training and the elaboration and improvement of clinical guidelines, facilitating the proper management of the health of the elderly, preventing avoidable hospitalizations and iatrogenesis in the treatment of morbidities [17, 19]. This study therefore aims to measure the prevalence of multimorbidity and its association with demographic, socioeconomic and health care variables in the elderly population. Observed and expected dyads and triads of diseases were also estimated.

## Methods

A population-based cross-sectional study was performed using data collected between July and November 2008 on individuals aged 60 or older, resident in the primary health care service catchment area of the urban zone of the municipality of Bagé-RS, located on the southern border of *Rio do Grande do Sul* state with Uruguay. In 2008 Bagé had some 120,000 inhabitants (84 % in the urban zone). The Family Health Strategy (FHS) had been implanted for five years and covered 51.0 % of the urban population. The rest of the population was covered by the traditional health care model. The elderly accounted for approximately 14 % of the population.

The sample size was calculated for a larger study [20, 21]. Considering 10 % for losses and refusals, as well as a design effect of 1.3, the study had 80 % statistical power to detect relative risks of 1.5 and exposures affecting at least 4 % of population.

When delimiting the sample, the catchment area of each of the PHC centers was defined and later divided into micro-areas, with each block of buildings being numerically identified. To guarantee equiprobability at the household level, the starting point for data collection in each of the blocks was selected randomly. One in every six households were visited. No replacements were admitted. All residents of these households aged 60 or older were invited to take part in the study. Interviews not conducted after three attempts on different days and times were considered to be losses/refusals. The probability of locating an elderly household member was estimated at 1 in every 3 households. This estimate ensure a widespread distribution of the sample given the proportion of elderly individuals in the general population (8–10 %).

Data collection was done by 15 interviewers coordinated by three trained supervisors. Training included theoretical explanations about the questionnaire using an instruction manual, conducting interviews, practical training in field work logistics and meetings during the data collection stage. The interviews were conducted using structured questionnaires with pre-coded questions applied to all the elderly in the households selected. In cases of partial incapacity – elderly people with lucid and focused communication ability but needing everyday accompaniment –, family members and main carers provided the answers. Questions requiring self-reported answers were not applied in cases of total incapacity – elderly people unable to communicate and with complete dependence on family members and/or carers.

The outcome was multimorbidity measured according to the health problems presented in Table 1. Multimorbidity was operationalized through diseases count, and combining the diseases according to two cutoff points suggested in the literature: a. ≥ 2 morbidities; and b. ≥ 3 morbidities [1, 22, 23].

**Table 1:** Health problems used to operationalize multimorbidity

Morbidity	How information was gathered?	Question or scale	Case
1) High Blood Pressure (HBP)	Medical diagnosis self-reported	Has a physician told you that you have High Blood Pressure?	Yes
2) Diabetes	Medical diagnosis self-reported	Has a physician told you that you have diabetes or high blood sugar levels?	Yes
3) Lung problem	Medical diagnosis self-reported	Has a physician told you that you have lung problem (bronchitis, emphysema, COPD, asthma)?	Yes
4) Heart problem	Medical diagnosis self-reported	Has a physician told you that you have heart problem?	Yes
5) Stroke	Medical diagnosis self-reported	Has a physician told you that you have had stroke?	Yes
6) Rheumatism, arthritis or arthrosis	Medical diagnosis self-reported	Has a physician told you that you have rheumatism, arthritis or arthrosis?	Yes
7) Disease in spinal column (any problem reported)	Medical diagnosis self-reported	Has a physician told you that you have a disease in your spinal column?	Yes
8) Cancer	Medical diagnosis self-reported	Has a physician ever told you that you had cancer?	Yes
9) Kidney problem	Medical diagnosis self-reported	Has a physician told you that you have a kidney problem?	Yes
10) Cognitive impairment	Scale	Mini-Mental State Examination (MMSE), composed of 30 items [46, 47]	≤22
11) Depression	Scale	Geriatric Depression Scale (GDS), composed of 15 items [48]	≥6
12) Urinary incontinence	Self-reported	Do you have problem of accidentally wetting yourself?	Yes
13) Amputation in any part of the body	Self-reported	At any time in life have you had to amputate some part of your body?	Yes
14) Eyesight problem	Self-reported	Does your eyesight hinder you in doing the things you need or want to do?	Yes
15) Hearing problem	Self-reported	Does your hearing hinder you in doing the activities that you need or want to do?	Yes
16) Problem chewing food	Self-reported	Do you have any problem or difficulty chewing food?	Yes
17) Falls	Self-reported	Have you fallen at any time since <1 year ago > until now?	Yes

The demographic, socioeconomic and health services variables included were: sex (male/female); self-reported skin color (white/black/yellow, brown or indigenous); age (60–64/65–69/70–74/≥75 years old); marital status (married or living with a stable partner/widow(er)/divorced or never married); years of schooling (none/1–7/≥8); economic class as per the *Associação Brasileira de Empresas de Pesquisas (ABEP)* (A and B – richer/C/D and E – poorer); bedridden in the month prior to the interview (no/yes); private health plan (no/yes); medical visit in the 3 months prior to the interview (no/yes); emergency services visit in the 3 months prior to the interview (no/yes); hospitalization in the twelve months prior to the interview (no/yes); and type of primary care center (traditional/FHS).

The proportions and their respective 95 % confidence intervals were calculated. The mean, median and interquartile range (Q25-Q75) were measured for the length of time the person had had the disease (diseases with medical diagnosis and amputation) and the number of diseases. In addition a projection of observed prevalence was made in order to estimate the absolute number of elderly people living in Bagé city with a given morbidity or multimorbidity. As such, the prevalence found was extrapolated to include all elderly people living in the urban area of the municipality of Bagé-RS in 2010 based on information available in the 2010 census conducted by the Brazilian Institute of Geography and Statistics (IBGE) (available at: http://cod.ibge.gov.br/6e6j).

The prevalence of dyads (≥10 %) and triads (≥5 %) of health problems was measured. The ratios (and respective 95 % confidence intervals) between observed and expected frequency were calculated to measure any occurrence of dyads and triads beyond expected frequency by chance [24]. The expected frequencies were calculated by multiplying individual prevalence of the diseases. Data analysis was performed using Stata version 12.

The project was approved by the Research Ethics Committee of the Federal University of Pelotas (Protocol No. 015/2008). Ethical principles were ensured using a Voluntary Informed Consent form signed by the respondents or those responsible for them. The right not to participate in the study and the anonymity of the respondents was guaranteed. The authors declared that they had no conflict of interest in this study.

## Results

We identified 1593 elderly household members. Losses represented 4.0 % and refusals, 3.0 %. Almost two thirds were women (62.8 %). The most reported skin color was white (78.6 %). Elderly people between 60 and 64 years old accounted for 25.1 % and those aged ≥75 accounted for 31.2 % of those interviewed. More than half (51.2 %) were married or lived with partner and 33.8 % were widowed. The majority of the elderly had between 1 and 7 years of schooling and 23.7 % had not attended school. Economic classes D/E and C accounted for 34.0 and 38.9 %, respectively. The bedridden represented 9.3 % of the sample. Two-thirds (35.4 %) have private health plan. More than a half (54.6 %) had a medical visit, 12.8 % visited emergency services and 17.7 % were hospitalized. The living in FHS catchment areas covered 53.5 % of the elderly (Table 2).

**Table 2:** Sample description and prevalence of multimorbidity according demographic, socioeconomic and health services characteristics

Variables	Sample		Multimorbidity			
	*n*	%	≥2 % (95 % CI)	No. of cases in target population	≥3 % (95 % CI)	No. of cases in target population
Sex						
Male	593	37.2	67.3 (63.4; 71.3)	3795	45.9 (41.7; 50.1)	2589
Female	1000	62.8	82.1 (80.0; 84.6)	7816	65.3 (62.3; 68.4)	6217
Skin color						
White	1252	78.6	74.7 (72.2; 77.2)	8901	55.1 (52.2; 57.9)	6566
Black	139	8.7	79.8 (72.7; 86.9)	1052	68.5 (60.3; 76.8)	903
Brown/yellow/indigenous	202	12.7	86.2 (81.3; 91.2)	1660	70.4 (63.8; 76.9)	1355
Age						
60–64	400	25.1	72.4 (68.0; 77.0)	2755	52.0 (46.9; 57.0)	1979
65–69	374	23.5	72.1 (67.5; 76.8)	2569	55.4 (50.3; 60.6)	1974
70–74	322	20.2	77.7 (73.0; 82.5)	2379	57.8 (52.2; 63.4)	1770
≥ 75	497	31.2	83.3 (79.8; 86.8)	3940	66.1 (61.6; 70.5)	3126
Years of schooling						
None	372	23.7	87.1 (83.5; 90,7)	3129	72.1 (67.4; 76.9)	2590
1–7	858	54.5	76.9 (74.0; 79.9)	6354	57.3 (53.8; 60.7)	4734
≥ 8	342	21.8	64.8 (59.5; 70.0)	2142	45.3 (39.8; 50.8)	1497
Economic class (ABEP)						
A and B (richer)	429	27.1	69.1 (64.6; 73.7)	2839	51.3 (46.3; 56.2)	2108
C	615	38.9	75.1 (71.6; 78.7)	4429	55.7 (51.6; 59.8)	3285
D and E	537	34.0	84.0 (80.8; 87.2)	4330	66.1 (62.0; 70.3)	3407
Bedridden						
No	1445	90.7	80.3 (78.1; 82.4)	11041	61.9 (59.4; 64.5)	8511
Yes	148	9.3	92.7 (88.1; 97.3)	1307	86.2 (80.1; 92.3)	1215
Private health plan						
No	1025	64.6	82.8 (80.4; 85.2)	8109	65.6 (62.6; 68.7)	6424
Yes	561	35.4	78.8 (75.3; 82.2)	4229	61.3 (57.1; 65.4)	3290
Medical visit						
No	723	45.4	73.2 (69.8; 76.6)	5038	54.1 (50.3; 57.9)	3724
Yes	868	54.6	87.9 (85.7; 90.2)	7276	72.1 (69.0; 75.2)	5968
Emergency services visit						
No	1387	87.2	79.4 (77.2; 81.6)	10496	61.4 (58.8; 64.1)	8117
Yes	204	12.8	95.1 (92.0; 98.2)	1845	82.1 (76.5; 87.6)	1593
Hospitalization						
No	1310	82.3	79.9 (77.6; 82.1)	9969	61.3 (58.6; 64.1)	7648
Yes	282	17.7	88.2 (84.3; 92.2)	2367	76.5 (71.2; 81.7)	2053
Primary Health Care						
Traditional	741	46.5	77.9 (74.8; 81.1)	5491	59.7 (56.0; 63.4)	4208
FHS	852	53.5	84.2 (81.6; 86.7)	6829	67.6 (64.3; 70.8)	5483
Total	1593	100.0	81.3 (79.3; 83.3)	12325	64.0 (61.5; 66.4)	9702

*%* prevalence, *CI* confidence interval

**Fig. 1:**
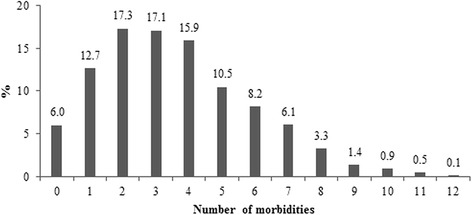
Prevalence of morbidities by number

The number of diseases in the same individual ranged from zero to 12 for the 17 problems listed in Table 1. Only 6.0 % of the total did not have morbidities. Less than 3 % had nine or more morbidities (Fig. 1). Mean morbidity was 3.6 (median = 3; Q25 = 2; Q75 = 5).

The percent of participants with multimorbidity was 81.3 % (95 % CI: 79.3; 83.3) for ≥2 morbidities and 64.0 % (95 % CI: 61.5; 66.4) for ≥3 morbidities. In both cases occurrence was higher among women, those with black or brown/yellow/indigenous skin, the more elderly, those with less schooling, less purchasing power, the bedridden, those who did not have a health private plan, those having had medical consultations and visited emergency services, those who had been hospitalized and those living in FHS catchment areas (Table 2).

Morbidities showing high prevalence were HBP (55.3 %) and spinal column disease (37.4 %), representing 8383 and 5670 elderly in the target population, respectively. Cancer (4.9 %) and amputation (3.5 %) were the least frequent conditions. Amputation and spinal column disease had been present for a longer length of time. The mean number of diseases ranged from 4.4 (HBP) to 5.8 (urinary incontinence and depression) (Table 3).

**Table 3:** Prevalence, cases number in target population, length of time with disease and number of diseases

Morbidities (*n*)	% (95 % CI)	No. of cases in target population	Time with disease^a^	Number of diseases
			Mean (median; Q25–Q75)	Mean (median; Q25–Q75)
High Blood Pressure - HBP (1593)	55.3 (52.9–57.8)	8383	10.6 (8; 3–15)	4.4 (4; 3–6)
Spinal column disease (1591)	37.4 (35.0–39.8)	5670	12.5 (10; 5–20)	4.7 (4; 3–6)
Cognitive impairment (1514)	34.1 (31.7–36.5)	5170	-	4.7 (4; 3–6)
Heart problem (1593)	29.6 (27.3–31.8)	4487	10.4 (8; 3–15)	5.3 (5; 4–7)
Falls (1591)	28.0 (25.8–30.2)	4245	-	5.1 (5; 3–7)
Eyesight problem (1547)	27.5 (25.3–29.8)	4169	-	5.3 (5; 4–7)
Rheumatism, arthritis or arthrosis (1592)	27.3 (25.1–29.5)	4139	10.9 (8; 3–15)	5.1 (5; 4–7)
Urinary incontinence (1592)	20.7 (18.7–22.7)	3138	-	5.8 (6; 4–7)
Problem chewing food (1580)	20.6 (18.6–22.6)	3123	-	5.3 (5; 3–7)
Depression (1512)	18.0 (16.1–19.9)	2729	-	5.8 (6; 4–7)
Diabetes (1593)	15.1 (13.4–16.9)	2289	7.7 (5; (2–10)	5.2 (5; 3–7)
Hearing problem (1550)	13.4 (11.7–15.1)	2031	-	5.3 (5; 4–7)
Stroke (1593)	9.9 (8.4–11.3)	1501	7.8 (5; 2–10)	5.7 (6; 4–7)
Lung problem (1593)	9.4 (7.9–10.8)	1425	13.3 (6; 2–15)	5.4 (5; 4–7)
Kidney problem (1591)	7.2 (6.0–8.5)	1092	12.1 (6; 2–15)	5.7 (5; 4–7)
Cancer (1591)	4.9 (3.8–6.0)	743	-	5.0 (5; 3–6)
Amputation (1582)	3.5 (2.6–4.5)	531	18.7 (14; 6–30)	4.8 (4; 3–6)

*%* prevalence, *CI* confidence interval, *Q25-Q75* interquartile range, *n* sample size

^a^Only for morbidities with medical diagnosis or for amputation

We found 22 dyads of morbidities with prevalence ≥10 % and 35 triads with prevalence ≥5 % (Tables 4 and 5). The most prevalent dyads of morbidities were HBP and spinal column disease (23.6 %), and HBP and heart problems (22.3 %). Four of the dyads did not have frequency statistically higher than expected by chance (Table 4). In the triads, this only occurred with the HBP/spinal column disease/cognitive impairment triplet (Table 5). In the dyads, the highest ratio between observed and expected frequency was found in rheumatism/arthritis/arthrosis and spinal column disease (O/E: 1.58–95 % CI: 1.43; 1.74) (Table 4). Regarding the triads, the highest prevalence found were HBP, rheumatism/arthritis/arthrosis and spinal column disease (10.6), and HBP, heart problem and spinal column disease (10.4 %). The highest ratio between observed and expected frequency related to triplet rheumatism/arthritis/arthrosis, spinal column disease and urinary incontinence (O/E: 2.53–95 % CI: 2.06; 3.10) (Table 5).

**Table 4:** Frequent co-occurring dyads (≥10 %) and observed and expected values

Frequent co-occurring dyads (*n*)	Observed (%)	Expected (%)	Observed/expected	95 % CI
HBP/spinal column disease (1591)	23.6	20.7	1.14	1.06–1.23
HBP/heart problem (1593)	22.3	16.4	1.37	1.26–1.48
HBP/cognitive impairment (1514)	19.9	18.9	1.05	0.97–1.15
HBP/eyesight problem (1547)	17.1	15.2	1.13	1.03–1.23
HBP/rheumatism (1592)	17.0	15.1	1.13	1.03–1.23
HBP/falls (1591)	16.8	15.5	1.09	1.00–1.19
Rheumatism/spinal column disease (1590)	16.1	10.2	1.58	1.43–1.74
Heart problem/spinal column disease (1591)	12.9	11.1	1.17	1.06–1.30
Spinal column disease/falls (1589)	12.9	10.5	1.23	1.11–1.37
HBP/urinary incontinence (1592)	12.8	11.4	1.11	1.01–1.23
Spinal column disease/eyesight problem (1545)	12.4	10.3	1.20	1.08–1.34
HBP/problem chewing food (1590)	11.5	7.4	1.55	1.38–1.75
Heart problem/cognitive impairment (1514)	11.4	10.1	1.13	1.01–1.26
Cognitive impairment/falls (1512)	11.3	9.5	1.18	1.06–1.33
Cognitive impairment/eyesight problem (1506)	11.2	9.4	1.19	1.06–1.33
Spinal column disease/cognitive impairment (1512)	11.1	12.8	0.87	0.78–0.97
HBP/depression (1512)	11.0	10.0	1.11	0.99–1.24
HBP/diabetes (1593)	11.0	8.4	1.32	1.17–1.48
Heart problem/eyesight problem (1547)	10.7	8.1	1.32	1.17–1.48
Rheumatism/falls (1590)	10.4	7.6	1.37	1.21–1.54
Eyesight problem/falls (1545)	10.4	7.7	1.34	1.19–1.52
Rheumatism/cognitive impairment (1513)	10.1	9.3	1.09	0.97–1.22

*%* prevalence, *CI* confidence interval, *n* sample size available to analysis

**Table 5:** Frequent co-occurring triads (≥5 %) and observed and expected values

Frequent co-occurring triads (*n*)	Observed (%)	Expected (%)	Observed/expected	95 % CI
HBP/rheumatism/spinal column disease (1590)	10.6	5.6	1.87	1.64–2.13
HBP/heart problem/spinal column disease (1591)	10.4	6.1	1.70	1.50–1.94
HBP/heart problem/cognitive impairment (1514)	8.8	5.6	1.58	1.37–1.81
HBP/heart problem/eyesight problem (1547)	8.6	4.5	1.91	1.65–2.21
HBP/spinal column disease/falls (1589)	8.6	5.8	1.48	1.29–1.69
HBP/spinal column disease/cognitive impairment (1512)	7.7	7.1	1.10	0.96–1.25
HBP/heart problem/falls (1591)	7.7	4.6	1.69	1.45–1.96
HBP/heart problem/rheumatism (1592)	7.7	4.5	1.73	1.49–2.01
HBP/rheumatism/falls (1590)	7.2	4.2	1.70	1.45–1.98
HBP/cognitive impairment/eyesight problem (1506)	7.1	5.2	1.37	1.18–1.59
HBP/rheumatism/cognitive impairment (1513)	6.8	5.1	1.33	1.14–1.54
HBP/cognitive impairment/falls (1512)	6.7	5.3	1.26	1.09–1.47
HBP/eyesight problem/falls (1545)	6.5	4.3	1.52	1.29–1.78
HBP/heart problem/urinary incontinence (1592)	6.5	3.4	1.91	1.61–2.26
Rheumatism/spinal column disease/falls (1588)	6.4	2.9	2.22	1.86–2.66
HBP/spinal column disease/urinary incontinence (1590)	6.2	4.3	1.45	1.24–1.70
HBP/rheumatism/eyesight problem (1546)	6.0	4.2	1.45	1.23–1.70
Rheumatism/spinal column disease/eyesight problem (1544)	6.0	3.5	1.71	1.44–2.03
HBP/heart problem/problem chewing food (1580)	5.9	3.4	1.77	1.49–2.10
HBP/diabetes/heart problem (1593)	5.9	2.5	2.39	1.97–2.88
HBP/cognitive impairment/urinary incontinence (1514)	5.9	3.9	1.50	1.27–1.78
HBP/cognitive impairment/depression (1502)	5.7	3.4	1.69	1.42–2.02
Heart problem/rheumatism/spinal column disease (1590)	5.7	3.0	1.87	1.57–2.24
HBP/depression/eyesight problem (1506)	5.6	2.7	2.04	1.68–2.46
HBP/urinary incontinence /eyesight problem (1547)	5.6	3.2	1.76	1.47–2.11
Heart problem/spinal column disease/eyesight problem (1545)	5.5	3.8	1.46	1.23–1.73
HBP/heart problem/depression (1512)	5.5	2.9	1.87	1.55–2.25
HBP/rheumatism/urinary incontinence (1591)	5.5	3.1	1.75	1.46–2.09
Rheumatism/spinal column disease/urinary incontinence (1589)	5.3	2.1	2.53	2.06–3.10
HBP/spinal column disease/problem chewing food (1578)	5.3	4.3	1.25	1.06–1.47
HBP/urinary incontinence /falls (1590)	5.3	3.2	1.64	1.38–1.96
HBP/eyesight problem/problem chewing food (1547)	5.2	3.1	1.67	1.39–2.00
HBP/spinal column disease/depression (1510)	5.2	3.7	1.41	1.18–1.67
HBP/depression/falls (1510)	5.0	2.8	1.80	1.49–2.19
HBP/cognitive impairment/problem chewing food (1514)	5.0	3.9	1.28	1.07–1.52

*%* prevalence, *CI* confidence interval, *n* sample size available to analysis

## Discussion

Multimorbidity frequency was high. At least 4 in every 5 major adults had ≥2 morbidities and 3 in every 5 had ≥3 morbidities, thus confirming the importance of multimorbidity as a frequent problem in older adults. The elevated number of dyads (*n* = 22) with prevalence ≥10 % and triads (*n* = 35) with prevalence ≥5 % highlights implications for the adequate management of health problems in the same individual, with HBP being the problem most often associated with other morbidities.

The percentage of multimorbidity found is consistent with the range of prevalence encountered in two systematic reviews [25, 26] and recent studies [27, 28]. When considering only population-based studies, the frequency found in our analysis was at least 10 percentage points higher with regard to the occurrence of ≥2 morbidities [25]. The comparability of multimorbidity studies is hampered owing to methodological differences, mainly related to the number of conditions included and the instruments used to measure morbidity.

Achieving standardization is a challenge to the development of knowledge about multimorbidity. References on this topic suggest that only chronic diseases should be included [1]. Despite the importance of acute conditions (e.g. influenza, tonsillitis and pneumonia), which are more susceptible to seasonal variations, their inclusion tends to inflate the occurrence of multimorbidity unnecessarily, thus complicating comparability [1]. Using at least 12 of the most prevalent morbidities appears to be advantageous because they showed lower variability in multimorbidity frequency [25]. Similar to the decision taken in this study, a recent review suggested the inclusion of certain geriatric syndromes in the construct of multimorbidity, such as urinary incontinence and falls [1], considering their relevance for the quality of life and independence of older people and for health care planning.

Therefore, taking the 12 most prevalent conditions in our data, multimorbidity frequency was 78.4 % (95 % CI: 76.4; 80.5) for ≥2 morbidities and 59.5 % (95 % CI: 57.0; 62.0) for ≥3 morbidities. After excluding urinary incontinence and falls from the morbidities selected in this analysis, the frequencies were 77.1 % (95 % CI: 75.0; 79.3) for ≥2 and 58.1 % (95 % CI: 55.6; 60.6) for ≥3 morbidities. These findings are slightly lower than those presented in results section. This reflects the low variability in the occurrence of multimorbidity in the sample, regardless of the selected conditions, and confirms the consistency of the prevalence found in this analysis.

The extrapolation of the data and its application to all the elderly living in the city of Bagé intend to subsidize the health policies at SUS, providing an opportunity for municipal health service management to plan actions for elderly people with multimorbidity. This analysis takes into account the percentage of older adults with given characteristics and this contributes to a more detailed evaluation to identify priority groups and the magnitude of impact for future interventions, thus allowing the adequate planning of actions aimed at these individuals. For example, the health care needs will be relatively higher among residents in FHS catchment areas compared to residents in traditional health service catchment areas in the city. Furthermore, the amount of older adults living in FHS are bigger, increasing their relevance for health planning. On the other hand, despite their low proportion, the management of multimorbidity may become more complex in the bedridden elderly compared to those who are not bedridden, eventually calling for more specialized care, and multidisciplinary teams.

Multimorbidity should not be seen as a major limitation of aging since its occurrence is more a rule than an exception. The complications and interactions of multiple chronic diseases represent a major challenge to the health services, because their impact on the autonomy and independence of individuals [17], increasing the risk of disability and frailty [29, 30]. Complications are related to exacerbation of chronic health problems, for example, uncontrolled high blood pressure that can lead to a stroke and increased risk of disability, or the lack of control of blood glucose levels generating micro and macrovascular problems closely related to the amputation of limbs.

The analysis by demographic, socioeconomic and health service type variables showed the profile of the individuals most affected by multiple problems. Higher occurrence among women may be attributed to survival bias since men tend to die earlier and those who survive are usually the healthiest [4]. Another explanation is related to greater use of medical services by females [31] which was also observed in this study (data not shown) thus enabling more opportunities for medical diagnosis of diseases. This results was similar to previous literature, including populations of others age groups [32, 33].

The more elderly who mentioned having black or brown/yellow/indigenous skin color had greater multimorbidity. This finding may be explained by the higher social and economic vulnerability of these individuals in Brazil, highlighting social inequities in health. The higher occurrence of multimorbidity among the more elderly is possibly justified by a greater exposition to physiological stress and, then, to the occurrence of chronic diseases.

The occurrence of multiple health problems was higher among older adults with less schooling and lower income. This finding is similar to the large majority of studies about multimorbidity [27, 33–35], reinforcing the social determination of health and disease. Furthermore, it is worth noting that Brazil is marked by inequalities in access to health services [15] and this could increases severity and complications.

The more elderly who used health services had greater multimorbidity. Reverse causality is marked in these associations because elderly people with more health problems may use more services or the use of services may have increased medical diagnosis. Nevertheless, these associations may reflect the importance of health service utilization as a marker of multiple chronic problems because, for example, almost all (95.1 %) the elderly who used emergency services had ≥2 health problems. Their relevance as a marker can be an efficient way of quick screening elderly people with multimorbidity during assessments by health professionals. Similarly, the bedridden elderly had more multimorbidity, reflecting the greater vulnerability of these individuals.

The associations with health private plans and the PHC model reflect the focus for actions directed towards management of multimorbidity. Elderly people without private plans and living in FHS catchment areas had more multimorbidity. This confirms social inequities since these elderly were poorer and less educated [21]. Whilst acknowledging that these actions may have been confused by socioeconomic indicators, we believe that an adjusted analysis would not make sense for the purpose of this article. Irrespective of confusion, individuals without a health plan and living in FHS catchment areas have more diseases and greater social and economic vulnerability. Thus, health actions related to the treatment and monitoring of chronic conditions should prioritize these individuals.

The observed/expected ratios were statistically insignificant in four dyads (HBP/cognitive impairment; spinal column disease/cognitive impairment; HBP/depression; and rheumatism/cognitive impairment) and one triplet (HBP/spinal column disease/cognitive impairment) with all having depression and cognitive impairment in the combinations. The measurement of these two conditions was done by screening tests, which can increase the false positive and reduce the specificity of combinations with causal relationship, as HBP and cognitive impairment [36]. All the other 18 dyads with prevalence ≥10 % and 34 triads with prevalence ≥5 % had a greater proportion than expected by chance. This reflects the occurrence of morbidity clusters and a possible causal relationship between morbidities and/or risk factors [37, 38].

However, the observed occurrence alone brings important information for clinical practice and management of the health system and health services in Brazil. For example, approximately one-fifth of the elderly have HBP and spinal column disease, thus indicating that activities for the proper management of a health problem should take into account all morbidities and not just one. For example, an elderly person with this pair of diseases should be well instructed on how to engage in physical activities, since although this is widely recognized as a good prognostic factor for HBP, it can also aggravate back problems if undertaken without adequate guidance [39]. The simultaneous occurrence of HBP and cognitive impairment was observed in same proportion as in the previous pair, thus highlighting the need for attention in the approach used in the pharmacological treatment of these elderly people. The same rationale is applicable to disease triads where morbidities and treatment interactions are more important and increase the complexity of health care management.

Worldwide, health systems are still unprepared for the management of individuals with multiple health problems and most guidelines are oriented towards a single disease despite the occurrence of multimorbidity [12, 40]. The evidence presented here – added to the findings in the international literature [38, 41] – contributes to guiding the development and adaptation of Brazilian clinical guidelines.

In order to overcome the challenge of multimorbidity, the current fragmented health care system for the elderly in Brazil should advance to a more comprehensive and multidimensional care [42]. Goals to tackle chronic conditions have recently been established with the publication of the strategic action plan to tackle NCD [14], the discussion on chronic care networks [13] and the consequent approval of the Ministerial Ordinance establishing the SUS Health Care Network for People with Chronic Diseases [43]. However, these guidelines do not adequately include multimorbidity, mainly owing to lack of information on the subject in the Brazil.

Promoting comprehensive care involving a considerable number of diseases, injuries, conditions and complications is a complex task, which requires similarly complex answers. The structure of a health system based on PHC is one of the leading measures to be taken by countries to reduce inequities and improve health care efficiency [44]. In Brazil, these efforts largely depend on FHS universalization and effectiveness.

Some limitations of this study should be addressed. Multimorbidity operationalization did not take into account the severity of the diseases, which could contribute to the identification of priorities in the appropriate management of multiple health problems. However, this approach would require greater detailing of disease severity and for the purpose of this study the use of disease counts is considered more useful than the use of scales/morbidities indices [45]. The other limitation is the absence of information about osteoporosis, thyroid disorders and dyslipidemia and the lack of adequate information necessary to characterize some morbidities. Although we have adequate measures for chronic morbidities through medical diagnosis (e.g. hypertension and diabetes) and screening for cognitive impairment and depression, we use proxies for other chronic morbidities, such as eyesight, hearing and oral health problems.

Among its strong points, this is a population-based study with low probability of selection bias in virtue of the low number of losses and refusals. Furthermore, the sample characteristics are similar to the Bagé and Brazilian census of elderly population collected in 2000 and 2010. These characteristics strength the internal and external inferences about study, providing support to policy-makers of Bagé and similar Brazilian cities in the actions related to multimorbidity. Moreover, the inclusion of a disease set affecting different body systems (e.g. circulatory, visual and urinary systems) have enabled a more complete approach to evaluate multimorbidity. Finally, reporting the findings in accordance with recommendations in the literature may have contributed to increasing comparability between studies.

## Conclusions

More findings on the prevalence of multimorbidity are needed in order to assess the problem in Brazil, given the scarcity of information. In addition, information about the complications and quality of care for individuals with multimorbidity will be key to ensuring the quality of life for people suffering from different chronic conditions.

Multimorbidity was high in the elderly in Bagé-RS, in keeping with percentages found in other countries. Characteristics of the population with a higher prevalence of multiple chronic problems revealed social inequities that are challenging the health services and health professional training to the adequate management of multimorbidity and its complications in Brazil.

## Abbreviations

ABEP: *Associação Brasileira de Empresas de Pesquisas*
FHS: Family Health Strategy
HBP: high blood pressure
IBGE: Brazilian Institute of Geography and Statistics
GDS: geriatric depression scale
MMSE: mini-mental state examination
NCD: noncommunicable chronic diseases
SUS: Sistema Único de Saúde (Unified Health System)
PHC: primary health care
RS: Rio Grande do Sul state
